# Immune Dysregulation in Children With Down Syndrome

**DOI:** 10.3389/fped.2020.00073

**Published:** 2020-02-27

**Authors:** Dean Huggard, Derek G. Doherty, Eleanor J. Molloy

**Affiliations:** ^1^Paediatrics, Trinity College, The University of Dublin, Dublin, Ireland; ^2^Trinity Translational Medicine Institute (TTMI), Trinity College Dublin, Dublin, Ireland; ^3^Paediatrics, Children's Hospital Ireland at Crumlin and Tallaght, Dublin, Ireland; ^4^National Children's Research Centre Dublin, Dublin, Ireland; ^5^Coombe Women and Infants University Hospital, Dublin, Ireland

**Keywords:** Down syndrome, immune dysregulation, immunodeficiency, review, innate immunity

## Abstract

Down syndrome (DS) is the most common genetic syndrome associated with immune defects. The extent of immune dysregulation in DS is substantial, spanning the innate and adaptive systems and including anomalies in: T and B cells, monocytes, neutrophil chemotaxis, circulating cytokines, and suboptimal antibody responses which all contribute to an increased risk of infections, poorer clinical outcomes and chronic inflammation in this vulnerable cohort. Other aspects of innate immunity may also be abnormal and contribute to the increased morbidity and warrant further interrogation such as: gamma delta T cell function, the inflammasome, Toll-like receptors and their pathways. Pharmacotherapies such as pavilizumab, pneumococcal and influenza immunizations, as well as potential immunoprophylactic agents such as pidotimod, azithromycin and Broncho-Vaxom may help alleviate the infectious consequences. Children with DS need to be managed with a heightened sense of awareness and urgency in the setting of sepsis and signs of chronic inflammation need regular screening and appropriate follow up.

## Introduction

Down syndrome (DS) is caused by extra genetic material from chromosome 21 and occurs in all ethnicities and across different species. It occurs in ~1 in 700 births in the USA, and 1 in 546 births in Ireland, which is the highest rate in Europe (1, 2). There are many co-morbidities associated with DS including; developmental delay, congenital heart disease, gastrointestinal anomalies, increased risk of hematological malignancy and several autoimmune conditions (3). It is also the most common genetic syndrome associated with immune deficits, with both the innate and adaptive responses being affected (4).

Children with DS are a high-risk group who get more severe infections and have poorer outcomes. In addition, they are more likely to be admitted to hospital, have an increased length of stay due to respiratory tract infection (RTIs) and a greater chance of requiring ventilatory support and intensive care (PICU) (5, 6). Infants with DS are more susceptible to severe respiratory syncytial virus (RSV) bronchiolitis with worse clinical outcomes and are more likely to be admitted to PICU with a higher overall mortality rate (7). Garrison et al. (8) reported that children with DS had a 30% increased mortality risk from sepsis than children without DS who also had sepsis. There is also evidence that their response to immunizations is sub-optimal, and that adaptive immunity may wane overtime contributing to their vulnerability to infection (9).

There are some anatomical considerations which may contribute to an increased predisposition to infection. Children with DS have a shorter midface and a relative macroglossia making it more difficult to clear secretions, leading to aspiration and LRTI development. Furthermore, they have a relatively short eustachian tubes which facilitates migration of pathogens into the middle ear (10), resulting in recurrent otitis media with effusion (OME) and sensorineural hearing loss (SNHL).

Autoimmune conditions such as hypothyroidism, coeliac disease, arthropathy and type 1 Diabetes mellitus are more prevalent in DS. These chronic inflammatory conditions are as a result of unchecked and persistent inflammation which can have significant long-term health complications (11). For example, in later life, this cohort represent the largest group of people with dementia under the age of 50 years (12). This neurodegenerative disorder is driven by aberrant neuroinflammatory processes which are exacerbated in DS (13), and may be driven by indolent chronic infection such as periodontitis, which is also more common in DS (14).

## T Lymphocytes

T lymphocytes are crucial parts of the adaptive immune system and are dichotomized into CD4+ or CD8+ based on their T cell receptor (TCR) and expression of CD4 or CD8. The former binds MHC class 1 molecules while CD8 interacts with MHC class 2 (15). During the first year of life there is normally a large expansion in the number of circulating T & B lymphocytes, however in DS there is an absence of these immunological changes (16). The fate of differentiating T and B cells over time is quite different in this population. de Hingh et al. (17) demonstrated that T lymphocyte numbers gradually increase toward the normal range over time, but B lymphocyte levels remain markedly reduced. These findings do point toward an inherent dysfunction of adaptive immunity in DS.

The thymus gland is a primary organ of lymphoid origin which is the site of T cell development and also has a crucial role in ensuring immune tolerance. Abnormalities of the thymus gland in DS including the smaller size of the organ have been known for some time (10). There is evidence of reduced T cell receptor excision circle (TREC) counts which represent recent emigrants from thymus gland and are a surrogate for T cell turnover (18). However, an appraisal of thymocyte development as well as Regulatory T cell (Treg) functionality has been less well-studied. Tregs are a key subtype of T lymphocytes which maintain self-tolerance and prevent autoimmunity by suppressing the immune system (19). Marcovecchio et al. (20) examined histological thymic samples from children with DS (*n* = 9), DiGeorge syndrome (DGS) (*n* = 10), controls (*n* = 26) and demonstrated that thymus in DS is hypocellular, smaller and with a reduced number of mature thymocytes. In the periphery there were reduced lymphocytes and Tregs which demonstrated decreased suppressive ability in patients with DS. These abnormalities may alter thymic selection of T lymphocytes and the Treg population leading to a greater propensity to develop autoimmune conditions.

In DS there are significantly reduced T lymphocyte numbers, both of CD4+ and CD8+ cells. Although absolute numbers will increase over time, deficient stimulation in response to circulating antigens may render the functionality and phenotype of these cells impaired (21, 22). Infants and children with DS have a reduced lymphocyte proliferative response to stimulation with phytohaemagglutinin (PHA) (23). Further evaluation of the function of T cell subpopulations on children and adults with DS (*n* = 40) and controls (*n* = 51), in response to pathogen specific stimulation with varicella zoster virus (VZV) and cytomegalovirus (CMV) found that the DS cohort could demonstrate an efficient effector T cell response with an equivalent phenotype and function to controls. However, the DS cohort needed greater effector T cell frequencies to eliminate pathogens (24). Noble et al. (25) reported a decrease in the number and functionality of helper T cells in children with DS cohort age matched controls. Furthermore, there may be an inherent defect in T helper cell responses to stimulation in DS in view of the normal levels of IL-2.

## B Lymphocytes

B lymphocytes are key players in all aspects of the adaptive immune response, they are derived from hematopoietic stem cells and following antigen presentation undergo proliferation, differentiation, and class switching to produce specific antibodies, and also retain memory to rapidly produce a high affinity response on subsequent encounter with the previous stimulating antigen (26). There are four subpopulations of B lymphocytes in peripheral blood; IgM memory B cells, switched memory B cells, mature naïve B cells and transitional B cells which have recently emigrated from the bone marrow (27). The switched memory cells are important as they represent the previous antigen experience of the individual and are imperative for an appropriate antibody response on encountering pathogens or following vaccination (28).

Further evidence of dysfunction of B lymphocytes in children with DS was demonstrated by Carsetti et al. (27) who found that DS is in fact a primary immunodeficiency disorder characterized by a fundamental defect in the differentiation of B cells leading to a significant decrease in switched memory B cells. These cells play a crucial role in the response to immunization and the secondary response to infectious organisms. The levels of immunoglobulins in DS are not profoundly different from controls. However, given their increased susceptibility to infection it can be argued therefore that switched memory B cells are important in the fight against infection and developing long term immunity post immunization, despite apparently normal serum immunoglobulins (29). Therefore, these cells are important in the response to vaccination and maintenance of adequate titers. Carsetti et al. demonstrated that transitional and mature naïve B cells are reduced by 50% in children with DS, and that switched memory B cells were lessened by 85–90% vs. controls. Although the total numbers of certain classes of B lymphocytes were found to be low, following stimulation with TLR-9 agonists children with DS mounted an exaggerated response and produced increased numbers of antibody generating cells from IgM and switched memory B lymphocytes. This demonstrated that children with DS can respond to antigenic stimulation.

There is conflicting evidence regarding serum immunoglobulin levels in DS. Valentini et al. (30) found that overall serum immunoglobulin levels were in the normal range, except for IgA which was found to be 40% lower compared with controls. They also found that salivary IgA was normal, despite the contrary being reported in other studies (31). Other research has shown adequate immunoglobulin levels in most children with DS (16, 32). Hypergammagloublinaemia of IgG and IgA after 5 years of age has been described in DS as well as decreased IgG2 and IgG4 and elevated IgG1 and IgG3(33). Despite relatively normal immunoglobulins in DS, the important clinical questions surround whether protection from pathogens is conferred and the maintenance of robust long term immunity.

A possible explanation for the observed differences in T and B lymphocyte number and function may be due to a tendency toward apoptosis in these cells. Apoptosis, also known as programmed cell death, is a cellular mechanism to remove unwanted cells. In the immune system it is important in eradicating poorly responsive B lymphocytes in germinal centers and deleting auto-reactive T lymphocytes in the thymus gland (34). There are reports that a propensity toward apoptosis in lymphocytes in children with DS may be a factor in the lymphopenias described. Gemen et al. (35) examined apoptotic markers (propidium iodide and Annexin V) by flow cytometry on peripheral lymphocytes in controls (*n* = 32) and children with DS (*n* = 72). There were greater levels of apoptosis in the DS cohort, which increased with age, and especially within B cells. This may be a cause for the reduced B lymphocytes seen in DS (36). Elsayed et al. (37) evaluated apoptosis by immunophenotyping and annexin V in 17 children with DS (*n* = 17) and controls (*n* = 17) found that there was also increased rates of apoptosis in DS, but contrary to the former publication, T cells were more profoundly affected. The authors concluded that it is the impairment of functionality in these cells that leads to the immune dysregulation and that cellular immunity was more markedly affected than humoral (37).

## Neutrophils

Neutrophils are a crucial effector cell, are the main phagocytes of the innate immune system and play an important role in clearing pathogenic micro-organisms (38). Izumi et al. (39) found significantly impaired neutrophil chemotaxis and periodontal disease in adults with DS vs. healthy controls (*n* = 14 in both groups) which they suggested may have a role in the poorer oral health of this population. Licastro et al. (40) found that phagocytic activity in children with DS (*n* = 27) was significantly decreased compared with controls (*n* = 23) which may point to an inherent defect in neutrophil functionality in DS. At the cell surface, receptors like CD11b (Mac-1) are important in the activation and migration of cells toward the site of infection or injury (41). Novo et al. (42) found no significant differences in CD11b expression on neutrophils in children with DS (*n* = 12, age 8–16 years) vs. controls (42). However, we reported a significant decrease in neutrophil CD11b at baseline in children with DS (*n* = 23, mean age 8.67 years) compared to controls (*n* = 21, age 7.4 years), and a significantly greater rise in CD11b post lipopolysaccharide (LPS; endotoxin) stimulation in the DS cohort vs. controls (43). This endotoxin hyperresponsiveness in neutrophils in DS which may lead to deleterious inflammatory consequences.

## Monocytes

The monocyte is another crucial innate immune cell that has several roles. They protect against foreign pathogens, clear dead cells, contribute to tissue repair and stimulate the adaptive immune system (44). Monocytes exist as a malleable, heterogeneous population and it is now accepted that there are three distinct subtypes, based on their relative CD14/CD16 surface positivity, which have distinct functions and are context dependent (45). The classical monocyte accounts for approximately 80% of the total monocyte population and expresses high levels of CD14 and is bereft of CD16 on their surface. The remainder have CD16 surface positivity and are separated based on the level of CD14 expression. More commonly the non-classical monocyte has very low CD14 and raised CD16 expression and the intermediate or inflammatory monocyte which has both cell surface markers increased (46).

The classical monocyte displays cell surface markers associated with antigen presentation, and the highest levels of CD163, CD36 which points to these cells having a major role in phagocytosis. The relative numbers are reduced in the setting of acute infection like sepsis or in chronic disease. The intermediate monocyte has multi functionality in phagocytosis and antigen presentation, but also in cytokine production such as Interleukin (IL)-10, increased Toll like receptor (TLR) cell surface expression and increased numbers in acute inflammation. We reported the highest baseline TLR4 (and TLR2) expression on intermediate monocytes in both children with DS and controls (43). The non- classical monocyte is pro-inflammatory and is the chief producer of pro-inflammatory cytokines IL-1β and Tumor Necrosis Factor (TNF-α) its numbers increase in both acute and chronic disease (47, 48).

Although pediatric research on monocyte subtypes in sepsis is limited, Skrzeczyñska et al. reported that infants (*n* = 30) had more CD14+/16+ and 14dim/16+ (intermediate and non-classical types), a reduced ability to phagocytose *E. coli in vitro* and produced less IFN-γ, IL-1, and more IL-10 (49). Monocytes in children with DS have similar anomalies. Bloemers et al. (50) examined the innate immune in DS children in detail and found that total leukocyte, lymphocyte and monocyte counts were decreased in the DS cohort compared to controls. Although total monocyte counts were reduced, there was a significant increase in the absolute number and overall percentage of non -classical or CD14dimCD16+ monocyte sub-population. Non-classical monocyte have been implicated in various disease states such as cancer, sepsis and chronic inflammation (51). We reported significantly elevated TLR-4 on non-classical monocytes in children with DS vs. controls (43), and greater TLR-2 expression on intermediate and non-classical sub-types (52). This portrays a pro-inflammatory phenotype of monocyte subpopulations in DS.

Functionality as well as overall monocyte numbers seem to be affected also in DS. One study involving 36 patients with DS and controls (*n* = 42), showed a significant reduction in monocyte chemotaxis (53). Khocht et al. found increased oxidative burst capacity from neutrophils and monocytes which correlated with clinical evidence of chronic inflammation and periodontitis (54).

## Natural Killer Cells

Natural killer (NK) cells arise from hematopoietic stem cells, and function in an effector and regulatory capacity. NK cells work by a combination of cytolysis and cytokine production (e.g., IFN-γ) and have anti-neoplastic, anti-viral and anti-bacterial actions (55). Bloemers et al. (50) reported a higher NK cell percentage in children from 1 to 9 years with DS (*n* = 41) vs. age-matched controls (*n* = 41) but not to a significant level. However, in the under 2-year olds a higher percentage of NK cells in the DS group was found (10.3 vs. 5.7% *p* < 0.01) (50). Maccario et al. (56) found a significant increase in the NK cell percentage in patients with DS (*n* = 25, *n* = 11; <10 years, *n* = 5 11–20 years, *n* = 9, 21–42 years) compared with controls (*n* = 25 age and sex matched) which did not increase with age. The functionality of NK cells in DS has been studied; Maccario et al. (56), described that NK cells in DS displayed a hypersensitivity to interferon stimulation. Cossariza et al. (57) evaluated numbers and function of lymphocytes in children (*n* = 10, average age = 9.2 years) and adults (*n* = 7, average age 43.2 years) with DS vs. age-matched controls found a significant increase in NK cell percentage in both DS groups. The proportion of NK cells increased dramatically in the adults with DS. There was a significant decrease in the cytotoxic activity in both DS age-groups compared with controls. The samples were also incubated with stimulatory cytokines IL- 2, IFN-γ, IFN-β, after which normal cytotoxicity was recorded, suggesting that in patients with DS, NK cells can respond to stimulation. There is conflicting evidence about the degree of aberrant NK functioning. Nurmi et al. (58) found deficient NK cell activity post stimulation with interferon-α in adults with DS compared with controls. Nair et al. (59) found that NK activity against target cells (K562) was reduced in DS and that the response to IL-2 was impaired vs. the control group. In contrast, there were no differences in the effects of Interferon alpha (IFN-α) on NK between children with DS and controls (60). Abnormal NK function adds to the evidence of a dysregulated innate immune system in DS.

## Gamma Delta (γδ) T Cells

The majority of the T lymphocyte population including CD4+ helper and CD8+ cytotoxic T cells express a CD3+ associated α/β T cell receptor. A smaller subset of T lymphocytes utilize heterodimeric T cell receptors composed of γ/δ chains (61). These γδ T cells have a varied tissue distribution in the body and are mostly enriched in several gastrointestinal and epithelial tissues, as well as in the epidermis. In peripheral blood they account for approximately 0.5–5% of the total lymphocyte count (62).

γδ T cells have a myriad of different functions and they are an important first line of defense from invading pathogens. They release several chemokines which increase neutrophil concentration at the site of infection and can also serve as antigen presenting cells, stimulate other adaptive and innate immune cells, while also retaining immunological memory. γδ T cells are key first responders to inflammation and propagate an early cytokine response (62). Cytokines such as IFN-γ, TNF-α, IL-17, and the anti-inflammatory IL-10 are known to be secreted by γδ T cells in the setting of autoimmunity or infection (63, 64).

There is a paucity of studies in the literature looking at γδ T cells in DS. Bertotto et al. (65) examined the proportion of blood lymphocytes bearing the γδ T receptor in this population and showed a significant increase in γδ T cells in adults with DS vs. controls. This was mainly attributed to a larger number of cells that express non-covalently bound γδ chains on their cell surface, in contrast to controls, where most of these cells had the disulphide-linked form of the receptor. These cells appear to also be different in number and perhaps function and are an important link between the innate and adaptive immune response.

## The Inflammasome

Inflammasomes are multi-protein complexes that generate IL-1 family cytokines. Their activation results in an innate inflammatory cascade involving caspases and the cleavage of pro IL-1β and IL-18 to their active forms (66). The NLRP3 inflammasome has been well-characterized and is associated with several medical conditions such as metabolic disorders, inflammatory bowel disease, multiple sclerosis and other autoinflammatory diseases (67). It is found mainly in innate immune cells, such as macrophages, dendritic cells, monocytes and neutrophils following inflammatory stimuli (68). As children with DS are reported to have significantly elevated levels of IL-1β (69), and are more prone to autoimmune conditions the inflammasome and its potential immunomodulation is potentially important target for further research in DS. Currently, there is a lack of research on the inflammasome in DS.

## Complement Pathway

The complement system is another critical component of innate immunity. Complement factor H (CFH) is secreted by the liver and after albumin is the most common plasma protein, and functions to inhibit conversion of C3 to C3b on the complement pathway. This results in dampening down and preventing spontaneous activation of the immune system. Deficiencies in CFH are associated with increased risk of persistent inflammation and autoimmunity (70). DS results in increased expression of certain genes and micro RNAs (miRNAs) on chromosome 21. miRNA-155 has been shown to be significantly increased in DS, and this causes a significant down-regulation of CFH mRNA which may partly explain the increased prevalence of chronic inflammation and autoimmunity in this population (71).

Alzheimer's disease (AD) is extremely common in DS, occurring with an earlier age of onset than the general population. The classical complement cascade and activation of the membrane attack complex in neurons in response to amyloid beta plaque deposition has been implicated in the development of AD in DS (72). Another study examining complement and AD in DS reported that C1q, which is the initial factor in the complement pathway, was increased in neurons with activated microglia and Abeta plaque accumulation, highlighting the importance of a dysregulated complement cascade in neurodegeneration (73).

There may indeed be a fundamental problem with the complement system in DS. Sullivan et al. (74) examined proteomics of blood samples from 263 people, 165 of whom had DS, and pointed toward an overall deficiency of complement factors or hypocomplementia as C1QA, C1R, C3 and C6 were downregulated. Indeed, hypocomplementia is associated with type 1 interferonopathies, which are also strongly associated with DS (75), suggesting that this persistent inflammation leads to the consumption of complement factors. Another clinical sequala associated with DS and reduced complement levels is otitis media and bacterial pneumonia (76).

## Toll Like Receptors

A key mechanism linking the innate and adaptive immune response is via Toll Like Receptor (TLR) signaling. TLRs are pattern recognition receptors (PRRs) located on the cell membrane cells including neutrophils, monocytes, macrophages, lymphocytes, dendritic and epithelial cells. They are located at the cell membrane where they can recognize and bind signal molecules. These molecules can be derived from microorganisms such as bacteria, viruses or fungi exhibiting pathogen associated molecular patterns (PAMPs e.g., LPS, peptidoglycan, flagellin) or from dying endogenous cells bearing damage associated molecular patterns (DAMPs e.g., heat shock proteins, oxidative stress) (77).

Activation of TLRs causes downstream signaling pathways which require a variety of five adaptor proteins. The Toll/interleukin-1 receptor (TIR) domain, which is found on the cytosolic face of both the TLRs and the adaptors is the main signaling area. The four remaining adaptor proteins involved in TLR downstream signaling are as follows: myeloid differentiation primary-response gene 88 (Myd88), MyD88-adaptor-like protein (MAL), TIR-domain-containing adaptor protein inducing interferon-β (TRIF), and TRIF-related adaptor molecule (TRAM). Their activation eventually results in increased production of the interferon regulatory factor (IRF) family and nuclear factor kB (NF- kB) (78), ultimately leading to inflammatory cytokine release.

Two important receptors involved in recognizing pathogenic ligands and maintaining host defense are TLR2 and TLR4, which predominantly bind to constituents of gram positive and gram-negative bacteria respectively (79). However, there are several studies showing that a dysregulation in these receptors can cause excess pro-inflammatory cytokines and chemokines, leading to autoimmunity, sepsis and multi-organ dysfunction (80, 81). These clinical sequelae are particularly relevant for children with DS. Infections from gram positive bacteria like *Streptococcus pneumoniae* and *Staphylococcus aureus* causing lower respiratory tract infections (LRTIs), and recurrent otitis media are more prevalent in children with DS and associated with poorer outcomes (5, 10). TLR2 is the major PRR involved in binding gram-positive bacteria and is strongly implicated in chronic inflammation. It is possible that dysregulation of this receptor and its pathways may be abnormal in DS, and our research on these immune signals supports this theory. Indeed, anomalous TLR2 signaling has been associated with unregulated pro-inflammatory cytokine production and autoimmunity (52, 82).

TLR4 is of interest as plays a key role in fighting infection, however its aberrant activation can also lead to excess pro-inflammatory cytokine release, persistent inflammation leading to septic shock and autoimmunity (83, 84). In mice with LPS-induced lung injury the benefits of utilizing TLR4 monoclonal antibodies to block the receptor have been observed. There was reduced inflammation and pulmonary oedema in those who had TLR4 attenuation (85). In adults with DS and periodontal disease compared to controls without periodontitis, there was no difference in the expression of TLR2 or TLR4 single nucleotide polymorphisms (SNPs) (86). We reported an increase in TLR4 expression on non-classical (CD14dim/CD16+) monocytes at baseline in children with DS vs. controls, highlighting an increase in pro-inflammatory phenotype in this cohort (43).

Dysregulation of TLRs can lead to excess pro-inflammatory cytokine release and damage to tissue, consequently appropriate regulation of TLR signaling is crucial in maintaining homeostasis. There are many regulators described for TLRs, with microRNAs (miRNAs) now being described as key controllers of signals from these receptors (87). O'Neill et al. (88), in their review of miRNAs and their influence on fine tuning TLR responses, described several key miRNAs that attenuate signaling. The following have been implicated in the control and reduction of TLR responses by manipulating transcription: Mal- miR-145, MyD88—miR-155, and TLR2—miR-105. TLR signaling is tightly controlled to prevent persistent inflammation with many negative regulators interacting at many levels of the TLR pathways to maintain a balance (89). These pathways may be aberrant in DS, and we demonstrated that MyD88 expression was significantly reduced and TRIF significantly increased compared with controls, suggesting perhaps a compensatory increase in MyD88 independent signaling pathway (52).

## Cytokines

One of the key outputs of immune cell activation is cytokine production. Cytokines are proteins secreted by various cells and result in specific communications and interactions between elements of the immune system (90). A regulated system is required to prevent chronic inflammation and autoimmunity and also to ensure an appropriate response to pathogenic insults. Indeed, if there is dysregulated pro and anti-inflammatory cytokine release in the setting of infection systemic inflammatory response syndrome (SIRS) and or compensatory anti-inflammatory response syndrome (CARS) may occur, which can lead to deleterious consequences for certain patients (91). Both pro and anti-inflammatory mediators were elevated in in a murine model of sepsis with early deaths and high Interleukin- 6, Tumor necrosis factor α (TNF-α), Macrophage inflammatory protein 2 (MIP-2), Interleukin 1 receptor antagonist (IL-1ra) predicted mortality within 24 h (92). In adult humans with abdominal sepsis, elevated pro and anti-inflammatory mediators were also associated with increased mortality (TNF-α, Interleukin- 8, Interleukin- 10, IL-1ra) (91). In a pediatric cohort admitted to PICU with influenza (*n* = 52), it was shown that significant early immune suppression (leucopenia, low TNF-α) was linked to concomitant *S. aureus* infection and death (93).

A large meta-analysis (19 papers, DS *n* = 957, Controls *n* = 541) examining circulating cytokines in children and adults in DS concluded that TNF-α, IL-1β, IFN-γ were significantly raised in DS (94). These mediators have been implicated in the development of chronic inflammation and autoimmune disease which are more common in our population of interest (95). Early onset Alzheimer's disease is another clinical feature of DS and IL-6 is a key cytokine associated with this neurodegenerative process (96). IL-10 serves to dampen down the inflammatory response by attenuating cytokines like IL-6 and TNF-α. There is evidence that IL-10 is elevated in DS, and it is hypothesized that the pronounced anti-inflammatory signals could be a contributor in the increased prevalence of respiratory tract infections and pneumococcal lung disease (32, 97). We found that at baseline children with DS had greater levels of both pro- (IL-2, IL-6) and anti-inflammatory cytokines (IL-10, IL-1ra), as well as other mediators (Epo, VEGF, GM-CSF) (98). This demonstrates a cohort exhibiting both a potent pro and anti-inflammatory phenotype which again may contribute to the worse outcomes in sepsis and the increased prevalence of chronic disease and autoimmunity. Pulmonary hypertension is more common in DS, and the excess Epo and VEGF we describe may be a contributory factor in the development of this disorder in these children (99).

## Vaccine Response

The response to vaccination is varied in children with DS and there are numerous papers citing suboptimal immune responses in this cohort (9, 100–102). This may have significant clinical consequences for a high-risk cohort more prone to severe RTIs and hospitalizations from vaccine preventable diseases like influenza and pneumococcus (5, 6). There is also evidence that despite initial adequate titers, the immune response may wane over time and that long-term immunity in DS may not be preserved as well as controls (103). Therefore, through public health campaigns it is imperative that immunization against these pathogens is highlighted and delivered routinely, and that this vulnerable cohort is studied and followed over time to ensure robust/adequate immunity is maintained. Tailored vaccination programmes may need to be considered.

## Future Directions

Another vaccine preventable illness which disproportionately affects infants with DS is respiratory syncytial virus (RSV) bronchiolitis. This pathogen is the primary cause of this illness, which accounts for significant number of hospital admissions and deaths throughout the world each year (104). Infants with DS are at increased risk for more severe disease independent of congenital heart disease status; one systematic review and meta-analysis reported a 9-fold increased mortality and 8.7-fold increase in risk of hospitalization (105) for infants with DS, and another recently backed this up reporting significantly increased admissions, length of stay and ventilatory requirement, again independent of CHD (106). A passive from of immunization is available via a monoclonal antibody to RSV, pavilizumab, which reduces burden of disease and admission rate by 55–72% (107). Currently, most countries do not offer this prophylaxis against RSV for infants with DS on a routine basis unless they have concomitant risk factors like CHD or prematurity. However, given the greater burden of RSV disease in this cohort; higher hospitalization rates, increased length of stay and requirement for PICU admission, regardless of CHD status, the evidence is growing to support universal vaccination for infants with DS (108–111).

Children with DS are a high-risk group who have more RTIs, are more likely to be hospitalized, and overall have worse clinical outcomes. Studies have taken place to assess the efficacy of various therapies in reducing the burden of infections in DS. In a systematic review undertaken by Manikam et al. (112), they report that an RCT comparing oral zinc with placebo did not show any real benefit for this cohort. La Mantia et al. (113) examined the immunostimulant pidotimod (3-L-pyroglutamyl-L-thiaziolidine-4 carboxylic acid, a synthetic dipeptide molecule) which acts by promoting pro-inflammatory cytokine release and phagocytosis, and reported a reduction in the severity and frequency of upper RTIs in children with DS (114). A recent meta-analysis of 29 RCTS (*n* = 4,344) concluded that pidotimod resulted in good efficacy and was safe in the management of recurrent RTIs in children (115).

A new immunomodulator Broncho-Vaxom is an oral therapy which is composed of lyophilized bacterial lysate from eight bacteria causing RTIs (*Haemophilus influenzae, Streptococcus pneumoniae, Klebsiella pneumoniae, Klebsiella ozaenae, Staphylococcus aureus, Streptococcus pyogenes, Streptococcus viridans*, and *Moraxella catarrhalis*). It promotes the immune response by increasing serum IgA and IgG as well as improving T lymphocyte stimulatory signaling (116). A meta-analysis appraising efficacy in pediatric populations in 53 RCTs (*n* = 4,851) reported that Broncho-Vaxom was efficacious in reducing recurrent RTIs in children and more large scale trials are required to evaluate its efficacy and safety further (117). Azithromycin is increasingly used in children with DS as prophylaxis against recurrent RTIs. To our knowledge no clinical trial solely examining children with DS and this agent has been undertaken to date, however, an RCT found that early treatment with Azithromycin in preschool children with a background of severe LRTIs, resulted in a significant decrease in the likelihood of severe LRTIs (118). This raises the prospect of the potential clinical benefits for children with DS in this context, and an RCT in this cohort would indeed be welcome to further assess this.

## Conclusion

**Figure 1:**
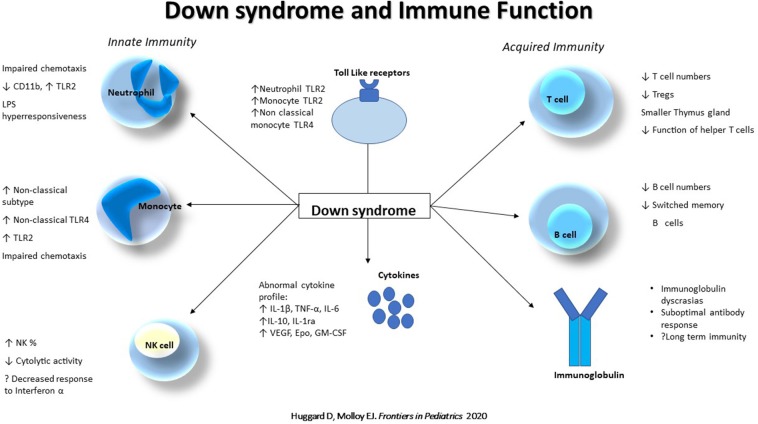
Down syndrome and immune function. Abnormalities of the innate (Neutrophil, Monocyte and Natural killer cell) and acquired (T and B cell, and immunoglobulins) immune system in Down syndrome. NK, Natural Killer cell; TLR, Toll like receptor; TNF, Tumor necrosis factor; Tregs, Regulatory T cells.

The extent of immune dysregulation in DS is substantial (Figure 1), spanning the innate and adaptive systems, anomalies in T and B cells, abnormal monocyte phenotype, neutrophil chemotaxis, circulating cytokines, and suboptimal antibody responses which contribute to a phenotype at risk of increased infections, poorer clinical outcomes and chronic inflammation. Other aspects of innate immunity may also be abnormal and contribute to the increased morbidity and warrant further interrogation such as: γδ T cell function, the inflammasome, TLRs and their pathways. Pharmacotherapies such as pavilizumab, pneumococcal and influenza immunizations, as well as potential immunoprophylactic agents such as pidotimod, azithromycin and Broncho-Vaxom may help alleviate the infectious burden. Consequently, these children need to be managed with a heightened sense of awareness and urgency acutely, in the setting of sepsis, and signs of chronic inflammation need regular screening and appropriate follow up.

## Author Contributions

DH was the principal researcher of the literature and author of the body of the manuscript. DD and EM provided further review of available evidence, contributed to writing, and editing of the manuscript.

### Conflict of Interest

The authors declare that the research was conducted in the absence of any commercial or financial relationships that could be construed as a potential conflict of interest.

## References

[1] Parker SE Mai CT Canfield MA Rickard R Wang Y Meyer RE. Updated National Birth Prevalence estimates for selected birth defects in the United States, 2004–2006. Birth Defects Res A Clin Mol Teratol. (2010) 88:1008–16. 10.1002/bdra.20735 20878909

[2] Ni She R Filan PM. Trisomy 21–incidence and outcomes in the first year, in Ireland today. Ir Med J. (2014) 107:248–9. 25282970

[3] van Trotsenburg AS Heymans HS Tijssen JG de Vijlder JJ Vulsma T. Comorbidity, hospitalization, and medication use and their influence on mental and motor development of young infants with Down syndrome. Pediatrics. (2006) 118:1633–9. 10.1542/peds.2006-1136 17015556

[4] Cruz NV Mahmoud SA Chen H Lowery-Nordberg M Berlin K Bahna SL. Follow-up study of immune defects in patients with dysmorphic disorders. Ann Allergy Asthma Immunol. (2009) 102:426–31. 10.1016/S1081-1206(10)60516-9 19492666

[5] Hilton JM Fitzgerald DA Cooper DM. Respiratory morbidity of hospitalized children with Trisomy 21. J Paediatr Child Health. (1999) 35:383–6. 10.1046/j.1440-1754.1999.00386.x 10457298

[6] Fitzgerald P Leonard H Pikora TJ Bourke J Hammond G. Hospital admissions in children with down syndrome: experience of a population-based cohort followed from birth. PLoS ONE. (2013) 8:e70401. 10.1371/journal.pone.0070401 23967074 PMC3742744

[7] Lee YI Peng CC Chiu NC Huang DT Huang FY Chi H. Risk factors associated with death in patients with severe respiratory syncytial virus infection. J Microbiol Immunol Infect. (2016) 49:737–42. 10.1016/j.jmii.2014.08.020 25442868

[8] Garrison MM Jeffries H Christakis DA. Risk of death for children with down syndrome and sepsis. J Pediatr. (2005) 147:748–52. 10.1016/j.jpeds.2005.06.032 16356424

[9] Joshi AY Abraham RS Snyder MR Boyce TG. Immune evaluation and vaccine responses in Down syndrome: evidence of immunodeficiency? Vaccine. (2011) 29:5040–6. 10.1016/j.vaccine.2011.04.060 21596078 PMC3909669

[10] Ram G Chinen J. Infections and immunodeficiency in Down syndrome. Clin Exp Immunol. (2011) 164:9–16. 10.1111/j.1365-2249.2011.04335.x 21352207 PMC3074212

[11] da Rosa Utiyama SR Nisihara RM Nass FR Oliveira NP Fiedler PT de Messias-Reason IT. Autoantibodies in patients with Down Syndrome: early senescence of the immune system or precocious markers for immunological diseases? J Paediatr Child Health. (2008) 44:182–6. 10.1111/j.1440-1754.2007.01229.x 17927730

[12] Ballard C Mobley W Hardy J Williams G Corbett A. Dementia in Down's syndrome. Lancet Neurol. (2016) 15:622–36. 10.1016/S1474-4422(16)00063-6 27302127

[13] Perluigi M Di Domenico F Buttterfield DA. Unraveling the complexity of neurodegeneration in brains of subjects with Down syndrome: insights from proteomics. Proteomics Clin Appl. (2014) 8:73–85. 10.1002/prca.201300066 24259517 PMC3965623

[14] Kamer AR Fortea JO Videla S Mayoral A Janal M Carmona-Iragui M. Periodontal disease's contribution to Alzheimer's disease progression in Down syndrome. Alzheimers Dement. (2016) 2:49–57. 10.1016/j.dadm.2016.01.001 27239536 PMC4879643

[15] Overgaard NH Jung JW Steptoe RJ Wells JW. CD4+/CD8+ double-positive T cells: more than just a developmental stage? J Leukoc Biol. (2015) 97:31–8. 10.1189/jlb.1RU0814-382 25360000

[16] Kusters MA Verstegen RH Gemen EF de Vries E. Intrinsic defect of the immune system in children with Down syndrome: a review. Clin Exp Immunol. (2009) 156:189–93. 10.1111/j.1365-2249.2009.03890.x 19250275 PMC2759463

[17] de Hingh YC van der Vossen PW Gemen EF Mulder AB Hop WC Brus F. Intrinsic abnormalities of lymphocyte counts in children with down syndrome. J Pediatr. (2005) 147:744–7. 10.1016/j.jpeds.2005.07.022 16356423

[18] Roat E Prada N Lugli E Nasi M Ferraresi R Troiano L. Homeostatic cytokines and expansion of regulatory T cells accompany thymic impairment in children with Down syndrome. Rejuvenation Res. (2008) 11:573–83. 10.1089/rej.2007.0648 18386990

[19] Kondelkova K Vokurkova D Krejsek J Borska L Fiala Z Ctirad A. Regulatory T cells (TREG) and their roles in immune system with respect to immunopathological disorders. Acta Med. (2010) 53:73–7. 10.14712/18059694.2016.63 20672742

[20] Marcovecchio GE Bortolomai I Ferrua F Fontana E Imberti L Conforti E. Thymic epithelium abnormalities in DiGeorge and Down syndrome patients contribute to dysregulation in T cell development. Front Immunol. (2019) 10:447. 10.3389/fimmu.2019.00447 30949166 PMC6436073

[21] Cocchi G Mastrocola M Capelli M Bastelli A Vitali F Corvaglia L. Immunological patterns in young children with Down syndrome: is there a temporal trend? Acta Paediatr. (2007) 96:1479–82. 10.1111/j.1651-2227.2007.00459.x 17727689

[22] Ferreira CT Leite JC Taniguchi A Vieira SM Pereira-Lima J da Silveira TR. Immunogenicity and safety of an inactivated hepatitis A vaccine in children with Down syndrome. J Pediatr Gastroenterol Nutr. (2004) 39:337–40. 10.1097/00005176-200410000-00007 15448421

[23] Rigas DA Elsasser P Hecht F. Impaired *in vitro* response of circulating lymphocytes to phytohemagglutinin in Down's syndrome: dose- and time-response curves and relation to cellular immunity. Int Arch Allergy Appl Immunol. (1970) 39:587–608. 10.1159/000230384 4250088

[24] Schoch J Rohrer TR Kaestner M Abdul-Khaliq H Gortner L Sester U. Quantitative, phenotypical, and functional characterization of cellular immunity in children and adolescents with Down syndrome. J Infect Dis. (2017) 215:1619–28. 10.1093/infdis/jix168 28379413

[25] Noble RL Warren RP. Altered T-cell subsets and defective T-cell function in young children with Down syndrome (trisomy-21). Immunol Invest. (1987) 16:371–82. 10.3109/08820138709087092 2961683

[26] Liang B Eaton-Bassiri A Bugelski PJ. B cells and beyond: therapeutic opportunities targeting inflammation. Inflamm Allergy Drug Targets. (2007) 6:142–9. 10.2174/187152807781696473 17897050

[27] Carsetti R Valentini D Marcellini V Scarsella M Marasco E Giustini F. Reduced numbers of switched memory B cells with high terminal differentiation potential in Down syndrome. Eur J Immunol. (2015) 45:903–14. 10.1002/eji.201445049 25472482 PMC4674966

[28] Capolunghi F Rosado MM Sinibaldi M Aranburu A Carsetti R. Why do we need IgM memory B cells? Immunol Lett. (2013) 152:114–20. 10.1016/j.imlet.2013.04.007 23660557

[29] Baumjohann D Preite S Reboldi A Ronchi F Ansel KM Lanzavecchia A. Persistent antigen and germinal center B cells sustain T follicular helper cell responses and phenotype. Immunity. (2013) 38:596–605. 10.1016/j.immuni.2012.11.020 23499493

[30] Valentini D Marcellini V Bianchi S Villani A Facchini M Donatelli I. Generation of switched memory B cells in response to vaccination in Down syndrome children and their siblings. Vaccine. (2015) 33:6689–96. 10.1016/j.vaccine.2015.10.083 26518399

[31] Chaushu S Yefenof E Becker A Shapira J Chaushu G. Severe impairment of secretory Ig production in parotid saliva of Down Syndrome individuals. J Dent Res. (2002) 81:308–12. 10.1177/154405910208100504 12097442

[32] Cetiner S Demirhan O Inal TC Tastemir D Sertdemir Y. Analysis of peripheral blood T-cell subsets, natural killer cells and serum levels of cytokines in children with Down syndrome. Int J Immunogenet. (2010) 37:233–7. 10.1111/j.1744-313X.2010.00914.x 20477881

[33] Nespoli L Burgio GR Ugazio AG Maccario R. Immunological features of Down's syndrome: a review. J Intellect Disabil Res. (1993) 37:543–51. 10.1111/j.1365-2788.1993.tb00324.x 8124000

[34] Koopman G Reutelingsperger CP Kuijten GA Keehnen RM Pals ST van Oers MH. Annexin V for flow cytometric detection of phosphatidylserine expression on B cells undergoing apoptosis. Blood. (1994) 84:1415–20. 8068938

[35] Gemen EF Verstegen RH Leuvenink J de Vries E. Increased circulating apoptotic lymphocytes in children with Down syndrome. Pediatr Blood Cancer. (2012) 59:1310–2. 10.1002/pbc.24246 22811045

[36] Verstegen RH Kusters MA Gemen EF E DEV. Down syndrome B-lymphocyte subpopulations, intrinsic defect or decreased T-lymphocyte help. Pediatr Res. (2010) 67:563–9. 10.1203/PDR.0b013e3181d4ecc1 20098344

[37] Elsayed SM Elsayed GM. Phenotype of apoptotic lymphocytes in children with Down syndrome. Immun Ageing. (2009) 6:2. 10.1186/1742-4933-6-2 19267926 PMC2657904

[38] Teng TS Ji AL Ji XY Li YZ. Neutrophils and immunity: from bactericidal action to being conquered. J Immunol Res. (2017) 2017:9671604. 10.1155/2017/9671604 28299345 PMC5337389

[39] Izumi Y Sugiyama S Shinozuka O Yamazaki T Ohyama T Ishikawa I. Defective neutrophil chemotaxis in Down's syndrome patients and its relationship to periodontal destruction. J Periodontol. (1989) 60:238–42. 10.1902/jop.1989.60.5.238 2525619

[40] Licastro F Melotti C Parente R Davis LJ Chiricolo M Zannotti M. Derangement of non-specific immunity in Down syndrome subjects: low leukocyte chemiluminescence activity after phagocytic activation. Am J Med Genet Suppl. (1990) 7:242–6. 10.1002/ajmg.1320370749 2149956

[41] Maiguel D Faridi MH Wei C Kuwano Y Balla KM Hernandez D. Small molecule-mediated activation of the integrin CD11b/CD18 reduces inflammatory disease. Sci Signal. (2011) 4:ra57. 10.1126/scisignal.2001811 21900205 PMC4507414

[42] Novo E Garcia MI Lavergne J. Nonspecific immunity in Down syndrome: a study of chemotaxis, phagocytosis, oxidative metabolism, and cell surface marker expression of polymorphonuclear cells. Am J Med Genet. (1993) 46:384–91. 10.1002/ajmg.1320460408 7689298

[43] Huggard D McGrane F Lagan N Roche E Balfe J Leahy TR. Altered endotoxin responsiveness in healthy children with Down syndrome. BMC Immunol. (2018) 19:31. 10.1186/s12865-018-0270-z 30390640 PMC6215672

[44] Boyette LB Macedo C Hadi K Elinoff BD Walters JT Ramaswami B. Phenotype, function, and differentiation potential of human monocyte subsets. PLoS ONE. (2017) 12:e0176460. 10.1371/journal.pone.0176460 28445506 PMC5406034

[45] Wong KL Yeap WH Tai JJ Ong SM Dang TM Wong SC. The three human monocyte subsets: implications for health and disease. Immunol Res. (2012) 53:41–57. 10.1007/s12026-012-8297-3 22430559

[46] Ziegler-Heitbrock HW. Heterogeneity of human blood monocytes: the CD14+ CD16+ subpopulation. Immunol Today. (1996) 17:424–8. 10.1016/0167-5699(96)10029-3 8854561

[47] Mukherjee R Barman PK Thatoi PK Tripathy R Das BK Ravindran B. Non-Classical monocytes display inflammatory features: validation in sepsis and systemic lupus erythematous. Sci Rep. (2015) 5:13886. 10.1038/srep13886 26358827 PMC4566081

[48] Ong SM Hadadi E Dang TM Yeap WH Tan CT Ng TP. The pro-inflammatory phenotype of the human non-classical monocyte subset is attributed to senescence. Cell Death Dis. (2018) 9:266. 10.1038/s41419-018-0327-1 29449647 PMC5833376

[49] Skrzeczyñska J Kobylarz K Hartwich Z Zembala M Pryjma J. CD14+CD16+ monocytes in the course of sepsis in neonates and small children: monitoring and functional studies. Scand J Immunol. (2002) 55:629–38. 10.1046/j.1365-3083.2002.01092.x 12028567

[50] Bloemers BL van Bleek GM Kimpen JL Bont L. Distinct abnormalities in the innate immune system of children with Down syndrome. J Pediatr. (2010) 156:804–9, 809.e1–5. 10.1016/j.jpeds.2009.12.006 20172534

[51] Ziegler-Heitbrock L. The CD14+ CD16+ blood monocytes: their role in infection and inflammation. J Leukoc Biol. (2007) 81:584–92. 10.1189/jlb.0806510 17135573

[52] Huggard D Koay WJ Kelly L McGrane F Ryan E Lagan N. Altered Toll-like receptor signalling in children with down syndrome. Mediators Inflamm. (2019) 2019:4068734. 10.1155/2019/4068734 31611734 PMC6757445

[53] Barroeta O Nungaray L Lopez-Osuna M Armendares S Salamanca F Kretschmer RR. Defective monocyte chemotaxis in children with Down's syndrome. Pediatr Res. (1983) 17:292–5. 10.1203/00006450-198304000-00013 6222283

[54] Khocht A Russell B Cannon JG Turner B Janal M. Oxidative burst intensity of peripheral phagocytic cells and periodontitis in Down syndrome. J Periodontal Res. (2014) 49:29–35. 10.1111/jre.12075 23488730 PMC3732561

[55] Herberman RB Nunn ME Holden HT Lavrin DH. Natural cytotoxic reactivity of mouse lymphoid cells against syngeneic and allogeneic tumors. II. Characterization of effector cells. Int J Cancer. (1975) 16:230–9. 10.1002/ijc.2910160205 1080480

[56] Maccario R Ugazio AG Nespoli L Alberini C Montagna D Porta F. Lymphocyte subpopulations in Down's syndrome: high percentage of circulating HNK-1+, Leu 2a+ cells. Clin Exp Immunol. (1984) 57:220–6. 6235075 PMC1536096

[57] Cossarizza A Ortolani C Forti E Montagnani G Paganelli R Zannotti M. Age-related expansion of functionally inefficient cells with markers of natural killer activity in Down's syndrome. Blood. (1991) 77:1263–70. 1825795

[58] Nurmi T Huttunen K Lassila O Henttonen M Sakkinen A Linna SL. Natural killer cell function in trisomy-21 (Down's syndrome). Clin Exp Immunol. (1982) 47:735–41. 6177458 PMC1536446

[59] Nair MP Schwartz SA. Association of decreased T-cell-mediated natural cytotoxicity and interferon production in Down's syndrome. Clin Immunol Immunopathol. (1984) 33:412–24. 10.1016/0090-1229(84)90312-x 6209046

[60] Noble RL Warren RP. Analysis of blood cell populations, plasma zinc and natural killer cell activity in young children with Down's syndrome. J Ment Defic Res. (1988) 32:193–201. 10.1111/j.1365-2788.1988.tb01405.x 2971114

[61] Holtmeier W Kabelitz D. Gammadelta T cells link innate and adaptive immune responses. Chem Immunol Allergy. (2005) 86:151–83. 10.1159/000086659 15976493

[62] Paul S Shilpi Lal G. Role of gamma-delta (γ*δ*) T cells in autoimmunity. J Leukoc Biol. (2015) 97:259–71. 10.1189/jlb.3RU0914-443R 25502468

[63] Duhindan N Farley AJ Humphreys S Parker C Rossiter B Brooks CG. Patterns of lymphokine secretion amongst mouse gamma delta T cell clones. Eur J Immunol. (1997) 27:1704–12. 10.1002/eji.1830270717 9247581

[64] Ashour HM Niederkorn JY. Gammadelta T cells promote anterior chamber-associated immune deviation and immune privilege through their production of IL-10. J Immunol. (2006) 177:8331–7. 10.4049/jimmunol.177.12.8331 17142729

[65] Bertotto A Scalise F Gerli R Castellucci G Fabietti GM Spinozzi F. Lymphocytes bearing the gamma/delta T-cell receptors in Down's syndrome. Scand J Immunol. (1992) 35:275–8. 10.1111/j.1365-3083.1992.tb02859.x 1531546

[66] Gross O Thomas CJ Guarda G Tschopp J. The inflammasome: an integrated view. Immunol Rev. (2011) 243:136–51. 10.1111/j.1600-065X.2011.01046.x 21884173

[67] Shao BZ Xu ZQ Han BZ Su DF Liu C. NLRP3 inflammasome and its inhibitors: a review. Front Pharmacol. (2015) 6:262. 10.3389/fphar.2015.00262 26594174 PMC4633676

[68] Guarda G Zenger M Yazdi AS Schroder K Ferrero I Menu P. Differential expression of NLRP3 among hematopoietic cells. J Immunol. (2011) 186:2529–34. 10.4049/jimmunol.1002720 21257968

[69] Broers CJ Gemke RJ Weijerman ME van der Sluijs KF van Furth AM. Increased pro-inflammatory cytokine production in Down syndrome children upon stimulation with live influenza A virus. J Clin Immunol. (2012) 32:323–9. 10.1007/s10875-011-9625-4 22170315

[70] Griffiths MR Neal JW Fontaine M Das T Gasque P. Complement factor H, a marker of self protects against experimental autoimmune encephalomyelitis. J Immunol. (2009) 182:4368–77. 10.4049/jimmunol.0800205 19299737

[71] Li YY Alexandrov PN Pogue AI Zhao Y Bhattacharjee S Lukiw WJ. miRNA-155 upregulation and complement factor H deficits in Down's syndrome. Neuroreport. (2012) 23:168–73. 10.1097/WNR.0b013e32834f4eb4 22182977 PMC3264826

[72] Stoltzner SE Grenfell TJ Mori C Wisniewski KE Wisniewski TM Selkoe DJ. Temporal accrual of complement proteins in amyloid plaques in Down's syndrome with Alzheimer's disease. Am J Pathol. (2000) 156:489–99. 10.1016/S0002-9440(10)64753-0 10666378 PMC1850044

[73] Head E Azizeh BY Lott IT Tenner AJ Cotman CW Cribbs DH. Complement association with neurons and beta-amyloid deposition in the brains of aged individuals with Down syndrome. Neurobiol Dis. (2001) 8:252–65. 10.1006/nbdi.2000.0380 11300721

[74] Sullivan KD Evans D Pandey A Hraha TH Smith KP Markham N. Trisomy 21 causes changes in the circulating proteome indicative of chronic autoinflammation. Sci Rep. (2017) 7:14818. 10.1038/s41598-017-13858-3 29093484 PMC5665944

[75] Sullivan KD Lewis HC Hill AA Pandey A Jackson LP Cabral JM. Trisomy 21 consistently activates the interferon response. Elife. (2016) 5:e16220. 10.7554/eLife.16220 27472900 PMC5012864

[76] Gross GN Rehm SR Pierce AK. The effect of complement depletion on lung clearance of bacteria. J Clin Invest. (1978) 62:373–8. 27534 10.1172/JCI109138 PMC371775

[77] O'Neill LA. The interleukin-1 receptor/Toll-like receptor superfamily: 10 years of progress. Immunol rev. (2008) 226:10–8. 10.1111/j.1600-065X.2008.00701.x 19161412

[78] O'Neill LA Bowie AG. The family of five: TIR-domain-containing adaptors in Toll-like receptor signalling. Nat Rev Immunol. (2007) 7:353–64. 10.1038/nri2079 17457343

[79] Redondo AC Ceccon ME Silveira-Lessa AL Quinello C Palmeira P Carvalho WB. TLR-2 and TLR-4 expression in monocytes of newborns with late-onset sepsis. J Pediatr. (2014) 90:472–8. 10.1016/j.jped.2013.12.012 24878008

[80] Meng G Rutz M Schiemann M Metzger J Grabiec A Schwandner R. Antagonistic antibody prevents toll-like receptor 2-driven lethal shock-like syndromes. J Clin Invest. (2004) 113:1473–81. 10.1172/JCI20762 15146245 PMC406529

[81] Williams DL Ha T Li C Kalbfleisch JH Schweitzer J Vogt W. Modulation of tissue Toll-like receptor 2 and 4 during the early phases of polymicrobial sepsis correlates with mortality. Crit Care Med. (2003) 31:1808–18. 10.1097/01.CCM.0000069343.27691.F3 12794424

[82] Drexler SK Foxwell BM. The role of toll-like receptors in chronic inflammation. Int J Biochem Cell Biol. (2010) 42:506–18. 10.1016/j.biocel.2009.10.009 19837184

[83] Tsukamoto H Fukudome K Takao S Tsuneyoshi N Ihara H Ikeda Y. Multiple potential regulatory sites of TLR4 activation induced by LPS as revealed by novel inhibitory human TLR4 mAbs. Int Immunol. (2012) 24:495–506. 10.1093/intimm/dxs053 22499954

[84] Rosadini CV Kagan JC. Early innate immune responses to bacterial LPS. Curr Opin Immunol. (2017) 44:14–9. 10.1016/j.coi.2016.10.005 27842237 PMC5426986

[85] He Z Chen X Wang S Zou Z. Toll-like receptor 4 monoclonal antibody attenuates lipopolysaccharide-induced acute lung injury in mice. Exp Ther Med. (2014) 8:871–6. 10.3892/etm.2014.1805 25120616 PMC4113535

[86] Oomori Y Imamura Y Fujigaki Y Hosaka K Miyazawa H Kasahara H Analysis of mutations of inflammatory cytokine and Toll-like receptor genes in periodontitis in Down syndrome patients. Pediatr Dent J. (2007) 17:19–26. 10.11411/pdj.17.19

[87] Caplan IF Maguire-Zeiss KA. Toll-Like receptor 2 signaling and current approaches for therapeutic modulation in synucleinopathies. Front Pharmacol. (2018) 9:417. 10.3389/fphar.2018.00417 29780321 PMC5945810

[88] O'Neill LA Sheedy FJ McCoy CE. MicroRNAs: the fine-tuners of Toll-like receptor signalling. Nat rev Immunol. (2011) 11:163–75. 10.1038/nri2957 21331081

[89] Kondo T Kawai T Akira S. Dissecting negative regulation of Toll-like receptor signaling. Trends Immunol. (2012) 33:449–58. 10.1016/j.it.2012.05.002 22721918

[90] Zhang JM An J. Cytokines, inflammation, and pain. Int Anesthesiol Clin. (2007) 45:27–37. 10.1097/AIA.0b013e318034194e 17426506 PMC2785020

[91] Surbatovic M Popovic N Vojvodic D Milosevic I Acimovic G Stojicic M. Cytokine profile in severe gram-positive and gram-negative abdominal sepsis. Sci Rep. (2015) 5:11355. 10.1038/srep11355 26079127 PMC4468818

[92] Osuchowski MF Welch K Siddiqui J Remick DG. Circulating cytokine/inhibitor profiles reshape the understanding of the SIRS/CARS continuum in sepsis and predict mortality. J Immunol. (2006) 177:1967–74. 10.4049/jimmunol.177.3.1967 16849510

[93] Hall MW Geyer SM Guo CY Panoskaltsis-Mortari A Jouvet P Ferdinands J. Innate immune function and mortality in critically ill children with influenza: a multicenter study. Crit Care Med. (2013) 41:224–36. 10.1097/CCM.0b013e318267633c 23222256 PMC3705720

[94] Zhang Y Che M Yuan J Yu Y Cao C Qin XY. Aberrations in circulating inflammatory cytokine levels in patients with Down syndrome: a meta-analysis. Oncotarget. (2017) 8:84489–96. 10.18632/oncotarget.21060 29137441 PMC5663613

[95] Tanaka T Kishimoto T. Targeting interleukin-6: all the way to treat autoimmune and inflammatory diseases. Int J Biol Sci. (2012) 8:1227–36. 10.7150/ijbs.4666 23136551 PMC3491446

[96] Licastro F Chiappelli M Ruscica M Carnelli V Corsi MM. Altered cytokine and acute phase response protein levels in the blood of children with Downs syndrome: relationship with dementia of Alzheimer's type. Int J Immunopathol Pharmacol. (2005) 18:165–72. 10.1177/039463200501800117 15698521

[97] Broers CJ Gemke RJ Morre SA Weijerman ME van Furth AM. Increased production of interleukin-10 in children with Down syndrome upon *ex vivo* stimulation with *Streptococcus pneumoniae*. Pediatr Res. (2014) 75:109–13. 10.1038/pr.2013.173 24126819

[98] Huggard D Kelly L Ryan E McGrane F Lagan N Roche E. Increased systemic inflammation in children with Down syndrome. Cytokine. (2019) 127:154938. 10.1016/j.cyto.2019.154938. 31785499

[99] Lemus-Varela ML Flores-Soto ME Cervantes-Munguia R Torres-Mendoza BM Gudino-Cabrera G Chaparro-Huerta V. Expression of HIF-1 alpha, VEGF and EPO in peripheral blood from patients with two cardiac abnormalities associated with hypoxia. Clin Biochem. (2010) 43:234–9. 10.1016/j.clinbiochem.2009.09.022 19804771

[100] Kusters MA Jol-Van Der Zijde EC Gijsbers RH de Vries E. Decreased response after conjugated meningococcal serogroup C vaccination in children with Down syndrome. Pediatr Infect Dis J. (2011) 30:818–9. 10.1097/INF.0b013e31822233f9 21849866

[101] Kusters MA Manders NC de Jong BA van Hout RW Rijkers GT de Vries E. Functionality of the pneumococcal antibody response in Down syndrome subjects. Vaccine. (2013) 31:6261–5. 10.1016/j.vaccine.2013.09.070 24200977

[102] Kusters MA Bok VL Bolz WE Huijskens EG Peeters MF de Vries E. Influenza A/H1N1 vaccination response is inadequate in down syndrome children when the latest cut-off values are used. Pediatr Infect Dis J. (2012) 31:1284–5. 10.1097/INF.0b013e3182737410 22986705

[103] Kusters MA Jol-van der Zijde CM van Tol MJ Bolz WE Bok LA Visser M. Impaired avidity maturation after tetanus toxoid booster in children with Down syndrome. Pediatr Infect Dis J. (2011): 30:357–9. 10.1097/INF.0b013e3181ff85a8 21057373

[104] Diaz-Diaz A Garcia-Maurino C Jordan-Villegas A Naples J Ramilo O Mejias A. Viral bacterial interactions in children: impact on clinical outcomes. Pediatr Infect Dis J. (2019) 38(Suppl. 1):S14–9. 10.1097/INF.0000000000002319 31205238 PMC6581203

[105] Beckhaus AA Castro-Rodriguez JA. Down syndrome and the risk of severe RSV infection: a meta-analysis. Pediatrics. (2018) 142:e20180225. 10.1542/peds.2018-0225 30093540

[106] Mitra S El Azrak M McCord H Paes BA. Hospitalization for respiratory syncytial virus in children with Down syndrome less than 2 years of age: a systematic review and meta-analysis. J Pediatr. (2018) 203:92–100.e3. 10.1016/j.jpeds.2018.08.006 30266507

[107] Gutfraind A Galvani AP Meyers LA. Efficacy and optimization of palivizumab injection regimens against respiratory syncytial virus infection. JAMA Pediatr. (2015) 169:341–8. 10.1001/jamapediatrics.2014.3804 25706618 PMC4391881

[108] Chan M Park JJ Shi T Martinon-Torres F Bont L Nair H. The burden of respiratory syncytial virus (RSV) associated acute lower respiratory infections in children with Down syndrome: a systematic review and meta-analysis. J Glob Health. (2017) 7:020413. 10.7189/jogh.07.020413 29302319 PMC5735780

[109] Stagliano DR Nylund CM Eide MB Eberly MD. Children with Down syndrome are high-risk for severe respiratory syncytial virus disease. J Pediatr. (2015) 166:703–9.e2. 10.1016/j.jpeds.2014.11.058 25596098

[110] Galleguillos C Galleguillos B Larios G Menchaca G Bont L Castro-Rodriguez JA. Down's syndrome is a risk factor for severe lower respiratory tract infection due to respiratory syncytial virus. Acta Paediatr. (2016) 105:e531–5. 10.1111/apa.13552 27537430

[111] Sanchez-Luna M Medrano C Lirio J. Down syndrome as risk factor for respiratory syncytial virus hospitalization: a prospective multicenter epidemiological study. Influenza Other Respir Viruses. (2017) 11:157–64. 10.1111/irv.12431 27611835 PMC5304568

[112] Manikam L Reed K Venekamp RP Hayward A Littlejohns P Schilder A. Limited evidence on the management of respiratory tract infections in Down's syndrome: a systematic review. Pediatr Infect Dis J. (2016) 35:1075–9. 10.1097/INF.0000000000001243 27273687 PMC5130062

[113] La Mantia I Grillo C Mattina T Zaccone P Xiang M Di Mauro M. Prophylaxis with the novel immunomodulator pidotimod reduces the frequency and severity of upper respiratory tract infections in children with Down's syndrome. J Chemother. (1999) 11:126–30. 10.1179/joc.1999.11.2.126 10326743

[114] Ferrario BE Garuti S Braido F Canonica GW. Pidotimod: the state of art. Clin Mol Allergy. (2015) 13:8. 10.1186/s12948-015-0012-1 25999796 PMC4440502

[115] Niu H Wang R Jia YT Cai Y. Pidotimod, an immunostimulant in pediatric recurrent respiratory tract infections: a meta-analysis of randomized controlled trials. Int Immunopharmacol. (2019) 67:35–45. 10.1016/j.intimp.2018.11.043 30530167

[116] Koatz AM Coe NA Cicerán A Alter AJ. Clinical and immunological benefits of OM-85 bacterial lysate in patients with allergic rhinitis, asthma, and COPD and recurrent respiratory infections. Lung. (2016) 194:687–97. 10.1007/s00408-016-9880-5 27117798 PMC7087659

[117] Yin J Xu B Zeng X Shen K. Broncho-vaxom in pediatric recurrent respiratory tract infections: a systematic review and meta-analysis. Int Immunopharmacol. (2018) 54:198–209. 10.1016/j.intimp.2017.10.032 29154122

[118] Bacharier LB Guilbert TW Mauger DT Boehmer S Beigelman A Fitzpatrick AM. Early administration of azithromycin and prevention of severe lower respiratory tract illnesses in preschool children with a history of such illnesses: a randomized clinical trial. JAMA. (2015) 314:2034–44. 10.1001/jama.2015.13896 26575060 PMC4757487

